# Identification of stress factors in returning migrants in Latvia

**DOI:** 10.3389/fpsyg.2024.1515406

**Published:** 2025-01-23

**Authors:** Iveta Ozola-Cīrule, Baiba Martinsone

**Affiliations:** Department of Psychology, University of Latvia, Riga, Latvia

**Keywords:** return migration, returnees, stress factors, qualitative interviews, Grounded Theory

## Abstract

This study investigates the psychological stress factors faced by return migrants before, during, and after their return to Latvia. Employing a Grounded Theory methodology, we conducted in-depth interviews with 21 return migrants and identified five key themes: pre-return context, identity, perceived social support, psychological wellbeing, and factors that help or hinder re-adjustment. Notably, psychological stress prior to return often exceeds post-return stress, highlighting the critical yet understudied pre-return phase. Key contributors to return migration stress include unmet expectations, feelings of alienation, identity struggles, and inadequate institutional support. By highlighting these stress factors, this research not only enhances the understanding of return migration from a psychological standpoint but also lays the foundational groundwork for the development of a comprehensive theoretical framework that encompasses a broader spectrum of factors influencing return migration stress. The study advocates for a holistic approach to supporting return migrants, emphasizing the integration of psychological resources with practical assistance to foster successful reintegration into their home country.

## 1 Introduction

Return migration research often emphasizes individual factors influencing the return process, including the importance of prior preparation (Cassarino, 2004), grief over leaving a life abroad (Butcher, 2002; Chamove and Soeterik, 2006), feelings of alienation (Fanari et al., 2021), and challenges in re-establishing cultural identity (Vathi and King, 2017), among others. However, these factors provide a fragmented explanation of the return experience for migrants and do not offer a comprehensive understanding of the challenges faced after prolonged absences. Return migration stress—also referred to as re-acculturative stress, re-entry stress, reverse culture shock, or return shock—describes the psychological and emotional difficulties experienced by return migrants. These challenges may include feelings of disorientation, anxiety, alienation, grief, and unexpected clashes with reality upon returning home, all of which can hinder the re-adjustment process (Szkudlarek, 2010). This stress arises from the complex interplay of situational, interpersonal, and cultural factors that shape the re-adjustment experience, highlighting the significant challenges return migrants encounter as they adapt to life back home (Černigoj et al., 2024).

Although existing studies have explored return migration stress (e.g., Vathi and King, 2017; Mohamed and Abdul-Talib, 2020; Szabo and Ward, 2023), a comprehensive understanding of stress factors remains incomplete, particularly within the field of psychology. In particular, the pre-return phase is still underexplored, emphasizing the need for further research into this critical aspect of the return migration experience.

In the first psychological study on return migration conducted in Latvia, Ozola-Cīrule and Martinsone (2023) found that half of return migrants experienced moderate to severe return shock. Similarly, a systematic review by Černigoj et al. (2024) reported that across 55 studies, 40–92% of participants faced at least moderate stress, with 12–24% experiencing high levels of stress. These findings indicate that returnees face significant psychological challenges, suggesting that psychology may be the missing link in understanding and addressing return migration difficulties.

Europe has faced significant population decline due to low fertility rates and increasing emigration. With a total fertility rate (TFR) of 1.4—well below the replacement level of 2.1—many European countries are experiencing natural population decreases (Population Reference Bureau, 2024). This trend is especially pronounced in Central and Eastern Europe, where emigration plays a major role. In Latvia, the population decline over the past three decades has been driven primarily by emigration, especially after joining the EU and the 2008–2009 economic crisis, resulting in a nearly 30% population drop (Hazans, 2019).

Like other Baltic states, Latvia has been cautious in implementing rapid immigration policies. While attracting immigrants could help mitigate depopulation, it introduces challenges such as integration issues and social tensions. In contrast, encouraging Latvian emigrants to return is generally viewed more favorably by the native population (Birka, 2019). Returnees often bring back valuable skills, knowledge, and experiences gained abroad, significantly contributing to the national economy and promoting local development. Although a recent increase in return migration has been observed, particularly during the COVID-19 pandemic, past efforts to attract return migrants have largely been ineffective (Kļave and Šūpule, 2019).

Latvia’s strategic focus should not only support return migration but also prioritize retaining those who have already returned. Data suggest that a significant portion—one-quarter—of returnees pursue repeat emigration (Hazans, 2019). While part of this trend is driven by transnationalism—the maintenance of active connections across national borders—some individuals leave again due to reintegration challenges linked to return migration stress factors.

Previous research has shown that returning is often more challenging than leaving home (Neuliep, 2015; Young, 2014). A recent volume, Yeo (2024), explores case studies highlighting how crises—such as economic downturns and political instability—shape the experiences of returnees. Combined with high levels of reported stress upon return and the issue of repeat emigration, the stress of return emerges as a significant theme deserving more in-depth research. A broader exploration of this issue aims to identify common stress factors, providing a more comprehensive understanding of the return process from a psychological perspective. Such an approach not only highlights the difficulties faced by return migrants but also offers valuable insights for developing targeted interventions to support smoother re-adjustment.

This paper makes an empirical contribution by identifying stress factors affecting return migrants through in-depth interviews, thereby laying the foundation for developing a theoretical framework that encompasses a broader range of factors associated with return migration stress. Based on this aim, the research question is: *What are the key stress factors reported by return migrants after their return to Latvia.*

## 2 Methodology

The Grounded Theory approach was employed to analyze the data obtained from the interviews. Developed by Glaser and Strauss, 1967, Grounded Theory is a qualitative research method designed to develop theory directly from data rather than from preconceived notions. It is particularly useful for exploring dynamic processes and constructing theoretical frameworks based on empirical findings (Corbin and Strauss, 2015). Grounded Theory emphasizes analyzing the progression and dynamics of a phenomenon, aiming to build a theory that emerges directly from the data.

The analytic process begins with a detailed examination of participants’ experiences. Through constant comparison, the data are systematically organized into abstract theoretical categories. This iterative process continues until a theory grounded in participants’ experiences emerges (Charmaz, 2014).

Grounded Theory is unique among qualitative research methods because concepts and theories are not predetermined but emerge as the research progresses. Additionally, data collection and analysis are interdependent, occurring simultaneously in a continuous cycle where each informs and shapes the other throughout the study (Corbin and Strauss, 2015). This approach allows for the refinement of emerging theoretical categories and the pursuit of new data as necessary, deepening the understanding of the phenomenon being studied.

### 2.1 Ethics approval

This study was conducted in accordance with the ethical guidelines issued by the Ethics Committee for Research in the Humanities and Social Sciences at the University of Latvia. The study received approval on March 14, 2024 (Approval No. 71–43/40).

### 2.2 Research participants

A total of 21 return migrants participated in the study. Participants were required to have lived abroad for a minimum of 2 years and to have returned to Latvia within the past 2 years. These criteria ensured that the study focused on individuals with substantial emigration experiences and recent re-adjustment processes (see Table 1). Beyond these requirements, no other specific inclusion criteria were applied.

**Table 1:** Participant sociodemographic data.

Code	Sex	Age	Host country	Time away (years)	Alone/family	Time since return (months)
Participant 1	F	40	Netherlands, France	15	Alone	10
Participant 2	M	35	Norway	2.5	Alone	10
Participant 3	F	26	United Kingdom	7	Partner	3
Participant 4	M	39	United Kingdom, Europe, Asia	13	Family	18
Participant 5	F	31	United Kingdom, Israel	8	Alone	24
Participant 6	F	37	Mexico	10	Family	24
Participant 7	F	32	France, Luxembourg, Spain	5	Alone	30
Participant 8	F	41	Canada, Australia, USA	20	Alone	30
Participant 9	F	31	United Kingdom	13	Family	3
Participant 10	M	37	United Kingdom	11	Family	2
Participant 11	F	36	United Kingdom	11	Family	2
Participant 12	F	7	Austria	7	Alone	11
Participant 13	F	36	Netherlands	11	Alone	9
Participant 14	F	26	Netherlands, Canada	2	Alone	5
Participant 15	F	36	United Kingdom, Germany	10	Children	10
Participant 16	M	70	United Kingdom	15	Alone	3
Participant 17	F	61	Germany	21	Alone	6
Participant 18	F	45	Africa and many other countries	15	Alone	24
Participant 19	M	48	United Kingdom	14	Alone	4
Participant 20	F	38	Germany	7	Family	12
Participant 21	F	42	Luxembourg, Germany, Belgium	20	Family	6

#### 2.2.1 Gender and age

The participants were 16 women and 5 men, with an age range from 26 to 70 years (*M* = 38.76, SD = 10.69).

#### 2.2.2 Countries of emigration and duration

Participants returned from various countries. The majority—eight participants—returned from the United Kingdom; three participants each returned from Germany and the Netherlands. Single participants returned from Austria, Canada, France, Mexico, Norway, and Luxembourg. Seven participants lived in three or more countries during their time abroad, contributing to the diversity of migration experiences. The duration of emigration ranged from 2 to 20 years (*M* = 11.31 years, SD = 5.35 years).

#### 2.2.3 Family status

Of the participants, 12 returned alone, while nine returned with their families. Among these families, eight had one to three children, predominantly of preschool age. Family status was considered to explore how social support, or its absence, relates to the stress of return.

#### 2.2.4 Time since return

The time since participants returned to Latvia varied between 2 months and 2 years (*M* = 11.71, SD = 9.39), allowing for an exploration of how stress changes over time as returnees readjust to life in their home country.

### 2.3 Procedure

Intensive interviewing was used to gather in-depth data on participants’ lived experiences (Charmaz, 2014), focusing on understanding the entire return process—from their decision to return, through their actions, thoughts, and emotions along the way, up to the point of the interview.

Participants were recruited between March and July 2024 through posts on social media platforms (Facebook and Twitter), community networks, and personal referrals based on their willingness to share their experiences. Interested individuals completed a Google form to provide their contact information for participation in the study. A total of 34 participants confirmed their participation; however, 13 either did not attend or canceled their scheduled interviews and did not reschedule. The remaining 21 participants were contacted via email to arrange interviews through the Google Meet platform at mutually agreed-upon dates and times. The interviews lasted approximately 1 h each.

Semi-structured interviews with open-ended prompts were conducted to capture participants’ return experiences without predetermined questions. This flexible approach allowed participants to focus on the aspects of their return they found personally significant, creating an open space for new insights to emerge.

At the beginning of each interview, participants were informed about the study, and informed consent was obtained. Sociodemographic information was collected, including age, country of emigration, length of time abroad, occupation, family status, and time since returning. The interviews typically began with the open-ended question: “Please tell me about your return experience, starting from the very beginning.” All participants began by discussing their reasons for emigrating and how they made the decision to return.

During the interviews, clarifying questions were posed to establish specific details or to request more in-depth commentary on certain topics. Toward the end of the interview, participants were invited to share any additional insights they felt were important but had not yet been discussed.

After compiling the data, some participants were contacted via email to clarify certain points or to provide additional information that emerged during the analysis. Their written responses were incorporated into the interview transcripts.

All interviews were recorded and later transcribed. The recordings were deleted once the transcripts were finalized. Participants were anonymized and assigned codes using a simple system such as “Participant 1,” “Participant 2,” and so on. During the interviews, the countries of emigration mentioned by participants were replaced in the transcripts with the term “host country.” This was done to prevent the identification of participants, since Latvia is a relatively small country and returnees from less common countries are rare, and to avoid associating the answers with any particular country.

### 2.4 Data analysis

The analysis followed the constructivist Grounded Theory guidelines stated by Charmaz (2014), employing an iterative approach that allowed for constant comparison and ongoing refinement. Data analysis began after the first interviews, with new data being collected and analyzed iteratively, facilitating adjustments throughout the research process. The analysis unfolded in three main phases: initial coding, focused coding, and theoretical integration.

*Initial coding* was conducted by coding texts line-by-line to derive a substantive number of codes. This approach ensured that no single theoretical account was settled on too early in the process.

After initial coding, *focused coding* was used to identify and refine the most significant categories from the data. This process condensed the initial codes into more abstract categories that encapsulated the core elements of the return migrants’ experiences. These categories provided a clearer structure for understanding the data and were essential for further analysis.

*Memo-writing* was integral for capturing reflections and analytical insights (Corbin and Strauss, 2015). This process aided in exploring relationships between categories, ensuring that the emerging theory remained grounded in the data.

In the final phase, *theoretical integration* synthesized the identified categories to develop overarching themes. These themes represented broader concepts that emerged from the data and captured the key aspects of return migrants’ experiences. Relationships between categories were examined to understand how they intersected, leading to the formation of a Grounded Theory that explained the core phenomenon under study.

The *constant comparative method* was applied throughout the analysis, with newly collected data continuously compared to existing categories for refinement. *Theoretical sampling* was employed by gathering additional data from the same participants to clarify and complete emerging categories, thereby enhancing understanding of return migration stress factors.

*Saturation* was reached when no new codes or themes emerged from the data (Corbin and Strauss, 2015), indicating that the analysis had sufficiently explored all relevant return migration stress factors.

*Trustworthiness* was ensured through applying criteria of credibility, originality, resonance, and usefulness (Charmaz and Thornberg, 2020). *Credibility* was maintained through systematic data collection and multiple rounds of coding. *Originality* emerged from the unique insights into the challenges return migrants face, contributing fresh perspectives on return migration stress factors within migration psychology. *Resonance* will be judged by return migrants and stakeholders, with findings shared through workshops and materials to enhance support systems. The study’s *usefulness* lies in its practical insights for improving return migration support programs, with a framework adaptable to other migration contexts.

Additionally, trustworthiness was enhanced by clearly articulating the rationale behind sampling design decisions, ensuring transparency in participant selection and criteria. Ethical considerations were thoroughly addressed, including obtaining informed consent, safeguarding participant anonymity, and adhering to established ethical guidelines. These efforts reflect the best practices for rigor and trustworthiness in qualitative research, as highlighted by Ahmed (2024).

Data were manually coded and organized systematically to ensure that all emerging categories were tracked and analyzed thoroughly. Manual coding facilitated a deeper engagement with the data, allowing for nuanced interpretations and flexibility in the analytical process.

## 3 Results

This section presents the findings from the analysis of interview data using a Grounded Theory approach. Five main themes emerged: (1) Pre-return context, (2) Identity, (3) Perceived social support, (4) Psychological wellbeing, and (5) Factors that help or hinder re-adjustment. These themes provide a comprehensive understanding of the stress factors faced by return migrants before, during, and after their return to Latvia.

### 3.1 Pre-return context

The Pre-return context theme encompasses the period leading up to participants’ return to Latvia, involving decision-making, emotional processing, and practical preparations. This phase was crucial, as participants often considered their return journey to begin well before their physical arrival (Table 2).

**Table 2:** Representative quotes from respondent interviews on pre-return context.

Theme	Category	Quotes from interviews
Pre-return context	Motivation to return	The COVID crisis in host country caused chaos and a sense of insecurity. During the pandemic, we were looking for safety, clarity, and trust in the government. Before COVID, we had not even considered returning; it wasn’t on our minds. However, we believed it would be easier to endure COVID in Latvia, where there was less chaos, and we felt safer.
		The arrival of our daughter was one of the factors that made us rethink our lives. When it was time to send her to daycare, we realized we did not want to do that in the host country. Family was our main reason for returning. We wanted to create memories for our daughter with her grandparents.
	Pre-return anxiety	After returning, the stress decreased. Once everyday life began, everything settled down, and things became calmer.
		The emotional aspect was extremely difficult, but the practical matters naturally fell into place.
	Farewell to the host country	I visited the old places where I used to live in the host country. It was interesting to return and reflect on how it was when I first arrived, how young I was, and how much I’ve grown. (…) I really wanted to visit the first place I lived. It was a symbolic farewell to that place. I took time for it and planned it out.
		I worked at a nursing home with people suffering from dementia. We had farewells. I knew it would be hard, but I wasn’t prepared for how many tears were shed; it was extremely emotional. I had never cried so much in my life during a farewell, all because of the emotions and the people.
	Preparation before return	Thorough preparation helped with settling in, as everything was well-organized and planned, with a good routine, so there was no need to worry about practical matters.
		It was important for me to discuss it with the children. I did not want to present it as a fact, but rather asked them if they could imagine it. What would be positive, and what would worry them? We pointed out the good things waiting for us there: cousins, extracurricular groups. We also talked about fears: what the school would be like, whether they would be able to make friends, how they would stay in touch with old friends, visiting them, and if something was difficult at school, they should say it right away so we could resolve it. Before moving, we all went together to visit the school to see what it looked like and met the teachers, so the children could see that the teachers were kind. We brought some of their favorite toys with us to Latvia. We also talked about how they would like to set up their new rooms, including things they’d always dreamed about, to make it feel pleasant. But still, there were shocks; you cannot prepare for everything easily, because changes just happen.
	Transition period	The period of isolation helps, as we have not yet met with friends or attended events. The sense of stability is gradually returning, and we are looking forward to the summer, when we can meet everyone, enjoy the culture, and go out more. In our own little bubble, it’s very peaceful and happy.
		In the first month, we took time off to relax. It was summer, warm, and we were setting up the house and hosting guests. The beginning was truly rosy.
		I had an internal crisis about what to do in Latvia. I needed time to figure out how to move forward. I did not want to live in Riga, but rather in the countryside. I wanted to be close to nature to help me settle in. During the first year, I did not do anything professional, I needed time to rest.

This table presents selected quotes from return migrants, illustrating their thoughts and experiences related to the period before their return.

#### 3.1.1 Motivation to return

Participants highlighted a variety of motivations behind their decision to return to Latvia. For many, the pull of family connections played a central role, as they sought to reconnect with relatives and raise their children in a Latvian cultural environment. Ensuring their children developed strong ties to their Latvian heritage was particularly important. The global instability brought on by the COVID-19 pandemic further influenced decisions, with Latvia being viewed as a safer and more secure option during uncertain times.

Homesickness and a longing for emotional fulfillment were also significant factors. Life events, such as having children, often shifted priorities toward re-establishing roots in their homeland. At the same time, dissatisfaction with their host countries—arising from feelings of alienation, unfavorable immigration policies, safety concerns, overcrowding, and dissatisfaction with the social and cultural environment—encouraged participants to make the move back to Latvia.

One participant shared:

*“Starting a family and having children has completely changed my perspective. I used to feel like a global citizen, traveling so much that I thought I could live anywhere. But having a family changed that feeling, and I decided I needed a place to put down roots.”*

#### 3.1.2 Pre-return anxiety

Participants reported significant anxiety *before* returning, often greater than the stress they experienced after returning. This anxiety stemmed from uncertainty, fear of failure, and doubts about their decision. The anticipation of the move and concern over potential challenges contributed to heightened emotional stress during this period.

*“When returning, our doubts were much greater than when we left. Comparatively, leaving was much easier.”*

#### 3.1.3 Farewell to the host country

Participants engaged in both real and symbolic farewells. Real farewells involved saying goodbye to friends and colleagues, while symbolic farewells included visiting favorite places for the last time. Participants who engaged in both real and symbolic farewells gained closure and prepared emotionally for the transition.

#### 3.1.4 Preparation before return

Approaches to preparation varied among participants. Some took a spontaneous approach, addressing practicalities after arrival. Others, particularly those with families, engaged in extensive planning. Preparations included securing housing and schools, discussing expectations, and emotionally preparing children for the move. Thorough planning facilitated smoother re-adjustment and reduced anxiety. However, the uncertainty of the return process was a recurring theme among participants, many of whom described limited preparation and a lack of clarity about what awaited them. One participant explained, *“We found a place to live and arranged to move our belongings, but we did not prepare much beyond that. We did not really know what to expect or what would require the most focus. It was a huge relief that my husband could keep his job.”*

#### 3.1.5 Transition period

Participants described the transition period after returning to Latvia as a mix of emotional highs and challenges. For some, the initial phase was marked by isolation and time spent adjusting slowly, often in a “bubble” with close family and familiar surroundings. Many participants took a break from work or school, allowing themselves time to rest, settle in, and adjust to their new environment. This phase typically lasted a few weeks, during which participants had not informed many people of their return. Instead, they focused on achieving inner stability and reflecting on their emotions, which helped ease the transition back into everyday life in Latvia. One participant described their experience: *“The beginning was completely crazy! The first month was spent in isolation because we did not want to meet anyone. Now, there’s a growing desire to meet other people and build connections. At this point, as we speak, the adjustment has only partially happened. We still only see the people we used to meet when visiting Latvia, but we’d like to meet new people as well.”*

### 3.2 Identity

The Identity theme explores how return migrants balanced their connections to both the host country and Latvia. Participants often viewed the host country as a “second home” while continuing to maintain strong ties to their Latvian roots. Returning sometimes led to an identity crisis, as they felt different from those who had never lived abroad. Many developed a fusion identity that blended both cultures (Table 3).

**Table 3:** Representative quotes from respondent interviews on identity.

Theme	Category	Quotes from interviews
Identity	Host country identity	We had a lot in common with people from the host country, and it was easier to understand them than Latvians. We also understood how everything worked in the host country. Now, we still feel a sense of alienation from our home environment. It’s difficult for us to predict how people will react here.
		I do not like Latvians—they hinder others from achieving more, do not help, and only think about themselves. In Latvia, bureaucracy, lack of transparency, order, fairness, and a proper system are frustrating, as is the lack of support and kindness. These were not issues in the host country. It’s also hard to accept the local materialism. All these factors make it difficult to fully integrate in Latvia. I’ll never feel as belonging here as I did abroad, where I felt accepted.
	Home country identity	When I thought about having a child, I imagined myself only in Latvia with my child—so that they would grow up in Latvia, be a part of this place, and know the Latvian language. I’m not a strong patriot, but for my child, I want that sense of belonging. When I think about having a child, these things become important.
		My friends who have left feel happy in their new countries. For me, it’s different—I feel like I’ve lost the ground beneath my feet. I sang in a [Latvian] choir in the host country, and the lyrics took on a completely different meaning. That’s when my Latvian identity became more pronounced.
		I never felt completely comfortable there; you are always a stranger. I realized that I do not like the culture of the host country and that I would never buy a house there.
		The crisis was a major motivator to feel a sense of belonging and attachment. The war [Russia’s war in Ukraine] and the nature of my work made it easy to bond with those around me. Having my own apartment, a home, also created a feeling of being in the right place.
	Identity crisis	I’m still trying to figure out how to find a job and settle in Latvia. At the same time, I’m also searching for my identity. I still feel like an outsider, unsure of where I belong. There’s still confusion, and it’s unclear where I should put down roots.
		I did not feel a sense of belonging abroad, and I thought I could find it in Latvia, but unfortunately, it’s not attainable here either.
	Fusion identity	I have not lived in just one country; the multinational environment has shaped a European sense of identity in me, rather than any specific culture. This openness allows me to develop my identity by connecting my Latvian roots with the international environment.
		I’ve noticed that both parts of my personality come through: with foreigners, one part of my personality is expressed, but with locals, I am completely different.
		I feel like a Latvian, but my experience abroad has had a strong impact on me, and I see those changes in myself since returning.

This table presents selected quotes from return migrants, illustrating their reflections on how living abroad and returning home has shaped their sense of identity.

#### 3.2.1 Host country identity

Participants expressed a strong connection to the host country, describing it as a place where they felt more accepted and understood than in Latvia. They found it easier to relate to people abroad, having adapted to the local culture and built a sense of belonging. They noted that the welcoming environment and openness of the host country made integration easier, fostering a feeling of being valued regardless of one’s profession or background.

The host country was where many participants experienced significant personal and professional growth and felt their identity fully formed. They viewed themselves as more aligned with the host country’s values and systems, especially when contrasting these with the challenges they perceived in Latvian society.

#### 3.2.2 Home country identity

Participants often described a deepening sense of connection to Latvia during their time abroad, with many expressing renewed pride in their roots. This deepening sense of national identity became more pronounced while living abroad, as they began to appreciate the unique aspects of Latvian culture, language, and values. Some felt a stronger desire to raise their children in Latvia, emphasizing the importance of passing on the Latvian language and traditions.

#### 3.2.3 Identity crisis

Participants frequently described experiencing an identity crisis upon returning to Latvia, as they struggled to reconcile their transformed sense of self with their new reality. Many had adapted to the culture and values of their host country, gaining confidence and professional experience. Upon returning, they felt undervalued and out of place in Latvia. They felt like they were starting from scratch, despite their education, skills, and international experience.

*“I also feel confused about my identity—who I am here, how I should feel, and what I should do. Right after returning, I felt that my personality had changed so much that I no longer wanted anything old from before I left. I felt pressure to make choices to preserve my new identity, to protect it from being threatened by the old things.”*

Feelings of alienation were common, with some participants expressing that neither Latvia nor the host country truly felt like home. This uncertainty about where they belonged contributed to a broader identity crisis as they sought to find their place personally and professionally. Some felt their personal growth and ambition were mismatched with the expectations or mindset in Latvia, causing internal conflict and frustration. The process of adjusting to Latvian society led some to feel they had to suppress their individuality or lower their expectations, which deepened the identity struggle.

#### 3.2.4 Fusion identity

Participants often developed a fusion identity, blending their Latvian roots with the cultural experiences of their host countries. Their time abroad shaped their worldview and personality, making them feel both Latvian and global citizens. Many appreciated the openness and flexibility they gained from international environments while still cherishing their Latvian heritage.

The host country remained a “second home,” fostering a lasting connection. This dual identity allowed them to combine aspects of both worlds, adopting traits from their host country while maintaining strong ties to Latvia. However, they often felt different from those who had never lived abroad, sensing that their experiences had set them apart. Despite this, they were able to navigate both cultures, creating an enriched and cohesive sense of self.

*“If I had not been away, I would not feel nearly as good as I do now. I’ve learned to appreciate and find value in everyday things within a broader context.”*

### 3.3 Perceived social support

The Perceived Social Support theme explores the wide range of support systems (or lack thereof) that return migrants encountered during their process of reintegrating into life in Latvia. This theme sheds light on how different aspects of personal relationships, social networks, and institutional frameworks are closely related to their transition back into the home country (Table 4).

**Table 4:** Representative quotes from respondent interviews on perceived social support.

Theme	Category	Quotes from interviews
Perceived social support	Their own family	It’s definitely easier to return with family because you can talk things through and share experiences with each other. If I did not have my family, I might not have returned. Moving alone would have been much harder.
		In our family, we sought support from one another. The experiences we went through together abroad have brought us closer; we have become used to relying on each other. If any problem arises, we can effectively resolve it within our family. Having shared so many experiences together strengthen the relationship and creates a harmonious partnership.
	Home country family	My parents eagerly awaited our return, which gave us the feeling of being truly welcomed. The sense of home is created by family, not the surroundings.
		In Latvia, I have my own bubble, and I have a very supportive family. If I did not have a good relationship with my family, I would not feel the same urge to return. Overall, my relationship with my family compensates for the negative feelings I get from other people in Latvia. My family is my safety net.
	Friends	During my time away, the friendships somehow faded. I did not have the energy to maintain them, and besides, in the host country, I quickly made new friends with whom I spent time locally. Upon returning, I did not feel the urge to reconnect. I guess it’s related to the fact that I feel significant changes within myself. Consequently, I also want different people around me.
		I invested so much in life in the host country and left behind friends with close relationships. I was angry with myself for not being able to maintain those close relationships with them now.
	Other return migrants	I had the support of family and close friends, who had also recently returned. I reached out to them, and we talked about what had happened. We concluded that we shared similar feelings.
		The association ‘With Global Experience in Latvia’ [return migrant organization] has helped me to settle in. I attend their events, and I like that I can meet people who are like me. There, I felt encouraged, and it’s also a place where information is exchanged.
	Society	I also realized that it would not be as comfortable as in the host country, for example, that society would not be as open and would be more conservative. I did not have high expectations about that, and I’ve experienced it—there are more negative emotions. In my own bubble, everything is fine, but the broader society is more hostile. I get the attitude that I’m from abroad and that I need to fit in and adapt
	Government, institutions, return migration coordinators	I got in touch with the coordinator. They are brilliantly useless. She suggested looking for information on Facebook. She did not have any useful information herself, but she was a nice girl. At Christmas, she sends tearful little poems, but what’s the point of that? I’m interested in entirely different information. It feels like they do not know what people need when they return.
		I relied on the state system, because if the state is honest and I’ve worked honestly, everything should be fine. But I got caught up in it. While living in host country, Latvia required tax declarations. I submitted them, even though people around me did not. But the law requires it, so I submitted the declarations. Latvia demanded its share of the taxes, even though there’s supposed to be double taxation avoidance. When I returned, the tax authority came down hard on me. I believe they have ruined the rest of my life.
		I wanted to apply for the one-time benefit. It seemed like I had to wait six months and then apply. When I applied, they told me that no, you must apply *within* six months. I got stubborn and tried to resolve it with a lawyer. That caused frustration. Everything was happening at the same time. I was deeply disappointed overall with the return migration policy.
		One of the shocks was the state of public safety, which I had not expected at all. I was shocked by the low level of public security. The attitude of the police was shocking. The level of violence is shocking. Being a woman in Latvia is different. Here, you must be more cautious and able to defend yourself.

This table presents selected quotes from return migrants, illustrating their experiences and reflections on the social support they received during their transition back to Latvia.

#### 3.3.1 Nuclear family

Participants frequently highlighted the importance of nuclear family support in facilitating their return to Latvia. Decisions were often made collectively, easing emotional and practical challenges; some noted that returning alone would have been far more difficult.

Families served as a primary source of emotional support, helping individuals navigate new experiences and challenges in Latvia. Shared experiences abroad strengthened family bonds, fostering trust and reliance on each other. Working together to solve problems and adjust to life in Latvia contributed to stronger relationships and smoother reintegration.

#### 3.3.2 Homeland family

Strong relationships with extended family in Latvia were key to reintegration for many participants, offering practical help and emotional support that eased the transition. Family provided a sense of belonging and stability, compensating for challenges in the broader social environment. However, for those without close family ties, the lack of support made the return more difficult and hindered their reintegration.

*“My family is nearby, but I would not say they were a source of psychological support. They helped practically, like taking care of the children, which is great in itself. But I did not burden them much with my emotional struggles—they probably would not understand.”*

#### 3.3.3 Friends

Participants noted that pre-emigration friendships often faded, as old friends no longer fully understood their experiences due to divergent life paths. As a result, they sought new social circles, particularly with others who had international experiences.

Some participants successfully built new friendships through work and social activities, while others found it more challenging to establish meaningful connections. Friendships maintained while abroad remained strong for some and served as an important support system upon their return. Many expressed that forming new friendships in Latvia was a gradual process requiring openness and time.

#### 3.3.4 Other return migrants

Many participants found significant support from fellow return migrants. Being around others with similar experiences provided comfort, a sense of community, and valuable advice during the reintegration process. Socializing with other return migrants through formal organizations or personal networks helped participants feel understood and accepted.

For some, these connections were vital, as they found it easier to bond with others who had faced the same challenges of returning to Latvia. Participants mentioned forming close friendships with returnees, attending events and support groups, and finding encouragement through shared experiences.

#### 3.3.5 Society

Participants frequently expressed frustration with Latvian societal attitudes, finding them more closed, conservative, and less supportive than those they experienced abroad. Some noted difficulty adjusting to less friendly behavior in public spaces and a more judgmental or unwelcoming environment. Others highlighted the shock of dealing with aggressive behavior in schools or feeling excluded from social circles due to their experiences abroad. One participant noted, *“I started being bothered by things that are taken for granted abroad but not in Latvia. People’s attitudes toward one another, like in public transport, where everyone always seems angry and sad. Also, practical things, like waste sorting.”*

Despite these challenges, a few participants explained that persistence and efforts to stay positive helped them shift interactions and form more meaningful connections. However, many felt the broader societal environment was less open and more rigid, limiting their ability to fully reintegrate into Latvian society.

#### 3.3.6 Government, institutions, and return migrant coordinators

Participants often encountered obstacles when dealing with government institutions and public services in Latvia. Many were disappointed with the lack of personalized support from return migrant coordinators, who often provided irrelevant advice. As one participant shared, *“I feel disappointed with the return migration coordinator because I still had to handle all the practical matters on my own.”* Some felt that remigration programs failed to address their real needs, and bureaucracy in areas such as education, healthcare, and tax policies was seen as a major barrier.

Frustration with local authorities and a lack of clear communication were common. Despite promises of support for return migrants, participants felt that the government’s efforts were inadequate, leading to disappointment and feelings of being unsupported during the reintegration process.

### 3.4 Psychological wellbeing

The Psychological wellbeing theme captures the emotional challenges that return migrants faced during their reintegration into Latvia. It encompasses distress, anxiety, feelings of loss, and a sense of alienation, often tied to unmet expectations. This theme highlights the deep emotional impact of the return journey beyond practical challenges (Table 5).

**Table 5:** Representative quotes from respondent interviews on psychological wellbeing.

Theme	Category	Quotes from interviews
Psychological wellbeing	Expectations and reality	I want to leave because I do not really feel at home here [in Latvia], but there’s nowhere to go there [in the host country] either, because it’s not home anymore. Feeling lonely abroad is okay, because it’s natural, but at home, there’s the expectation that you’ll feel good.
		I’ve changed, and the people here have changed too, which led to disappointment—there’s a great sense of loneliness here as well. When visiting, you are interesting to others, coming from an [exotic] host country. But in everyday life, it feels like no one is really interested in me. I feel like I’ve been thrown back and must start everything from scratch.
		I’m shocked that professionals aren’t needed in Latvia. Everyone is afraid of my experience. How can they not want to use it? It’s such narrow-mindedness, I do not understand it. After all, everyone should want growth.
	A sense of alienation	At first, I felt quite a bit of alienation. I did not feel like I belonged here, but I also did not feel a sense of belonging to the host country. It was a strange feeling at the beginning—lacking stability, attachment, and a sense of home. We largely cultivated that sense internally within the family to compensate for the discomfort.
	A sense of loss	The hardest part was saying goodbye to the garden—I stood there and cried. It’s a huge loss for me—the garden and the house.
		Spending time with my child is a loss [the child stayed with the mother in the host country]. I’m now a father from a distance. Physical closeness means a lot. I cannot go for walks or hug them. It’s emotionally heavy and difficult.
	Distress, anxiety	As a family, we all experienced anxiety. The youngest child could not accept that we had left. The older children withdrew into their screens and became distant, which had not been the case before. My husband was traveling back and forth, and it wasn’t easy for us to understand each other. I said that we did not need to be lectured; we needed help. We were all processing major changes. Everyone has their own pace of adjustment.
	Other emotions	It was important to me to succeed in settling in Latvia. I felt it as pressure that everything had to work out because I had to persuade my husband, and the children were taken out of their familiar environment. I felt guilty—mostly for my daughter, whose friendships fell apart. I also felt guilty for thinking only about myself.
		Upon returning, I was afraid of the changes that would come. I felt anxious. I was also afraid of how it would be for my family. I’m scared to start working because people in Latvia seem different. I’m afraid of how others will perceive us since we think differently than the locals. I’ve been afraid that we will not be accepted and will not be able to fit in here.

This table presents selected quotes from return migrants, reflecting their psychological struggles and emotional states during the process of return migration.

#### 3.4.1 Expectation and reality

This category highlights the gap between return migrants’ expectations and the reality they encountered upon returning to Latvia. While some found their expectations largely met, others faced unexpected challenges such as difficulties with the education system, housing, and professional barriers. Many experienced disappointment when confronting aspects of life in Latvia they had forgotten or hoped would change, including cultural differences, language barriers (some respondents, having lived abroad for up to 20 years, found it challenging to resume using Latvian in professional settings after becoming accustomed to their host country’s language), and social criticism. For some, these unmet expectations led to feelings of loneliness, uncertainty about belonging, and doubts about whether returning was the right decision.

*“Looking back now, I feel like I idealized things because it’s completely different after returning. Leaving was tied to a desire to escape the environment in Latvia, where I always felt criticized and not good enough. My inner critic is still very active here, but it wasn’t like that in host country. I feel it most in the professional field. The confidence I gained abroad is now diminishing in Latvia, creating a feeling that I have to start over, that my experience abroad has no value, that no one here needs me, and that no one appreciates me.”*

#### 3.4.2 A sense of alienation

The sense of alienation captures the feelings of isolation and disconnection many return migrants experienced upon returning. Despite being back in their homeland, many felt they did not fully belong, struggling to find their place or reconnect with social circles. Participants often felt like outsiders, uncertain about how to relate to local people or integrate into the community. This alienation was intensified by differences in mindset and behavior between themselves and those who had never lived abroad. Concerns about being misunderstood or not fully accepted by friends and family deepened their sense of dislocation. Despite attempts to reconnect and adapt, some continued to feel as though they were living between worlds—neither fully belonging in Latvia nor in the host country they left behind.

#### 3.4.3 A sense of loss

This category reflects the emotional struggles many return migrants faced as they came to terms with leaving behind their previous lives. Participants described feelings of loss not only for tangible aspects of their former homes, such as houses, gardens, and local amenities, but also for intangible elements like friendships, freedom, and cultural experiences. For some, the international environments they left provided a sense of belonging and openness that they now missed. The grieving process included letting go of familiar routines, opportunities for travel, and professional fulfillment. While some had made peace with their decision, many still carried lingering feelings of sadness and emotional attachment to their past life abroad, making it difficult to fully embrace their new reality in Latvia.

#### 3.4.4 Distress and anxiety

The distress and anxiety category captures the emotional toll that return migration placed on individuals and families. Many participants described overwhelming stress from the transition—not only from the move itself but also from challenges like re-entering the workforce, adjusting to new roles, and facing uncertainty about how their foreign experience would be valued in Latvia. Family dynamics were affected as children and spouses adapted at their own pace, adding to the emotional strain. For some, this prolonged stress resulted in burnout and other health issues as they navigated the complexities of their new reality.

*“The return itself was stressful, with prolonged stress until everything was organized and resolved. Very intense stress!”*

#### 3.4.5 Other emotions

This category encompasses the wide range of emotional experiences that return migrants underwent, including both positive and negative feelings. Participants described feelings of guilt, particularly regarding their families, as they navigated the impact of their return on children and spouses. Fears of the unknown, concerns about fitting in, or worries about finding work often compounded their stress. Some expressed a strong sense of responsibility and pressure to make the transition successful.

*“People are afraid to start something new, and they are also afraid to return. I lacked inner confidence; I was scared and ashamed of what others would think of me.”*

Positive emotions were also present—relief, hope, and excitement about rediscovering familiar places.

### 3.5 Factors that help or hinder re-adjustment

This theme highlights the personal, social, and practical elements that either eased or complicated the return migrants’ re-adjustment to life in Latvia (Table 6).

**Table 6:** Representative quotes from respondent interviews on factors that help or hinder re-adjustment.

Theme	Category	Quotes from interviews
Factors that help or hinder re-adjustment	Contributing factors	In the fall, there was a very beautiful period. Latvia’s national holidays were happening, and I went to everything, participated, trying to recapture that feeling. That helped me a lot—the shared activities people engage in. In the spring, the Kulakovs concert, that was wow! That’s when I understood why I had returned. The poppy seed rolls from Gustavs Bakery—nowhere else has anything like them—it’s such a wonderful feeling. The little things, like the cheese curds and rye bread, the Wednesday night market—all these small things have become rituals! My apple man in the market—we have become friends, and he’s already waiting for me to come back.
		Latvian food, nature, the sea, the peace, and the significantly smaller population. The large number of immigrants [in the host country] was very disruptive, and I felt like I was living outside of Europe. Right now, I view Latvia as more European.
		The place where we live now [in Latvia] is very green. You can climb a hill, my parents live in the forest, there are no neighbors, and you cannot find such an environment in the host country—your own little corner. In the host country, there’s no such privilege, just house after house with no free space.
		In the host country, our work hours were changed to align with Ramadan. There’s no national spirit there. I missed patriotism, like watching hockey, which unites the whole country. But there, individualism is so strong. People are lonely, and there’s a high suicide rate in schools. It’s hard to meet other people because of the different life rhythms; it’s a 24/7 industrial country where everyone is always working. I’m a single mother in Latvia, a teacher, and I was able to buy a small house. Over there, even two working people with good jobs cannot afford to rent an apartment. Here, there’s more money left over.
	Disruptive factors	In my work, I appreciate seeing different levels of development, and it’s painful to see how far behind Latvia has fallen. It creates a strong sense of injustice. Why do the people of Latvia, especially my parents’ generation, have to pay with their quality of life? Why cannot they enjoy the same level of prosperity as elsewhere, even though they have worked hard? I also see a sense of hopelessness among the youth—they want to leave and do not see a future for themselves here.
		The poverty in Latvia is unimaginable. All the elderly people, the little old ladies selling lilies of the valley—of course, I buy them. There’s a sense of hopelessness and helplessness. Just recently, in a tunnel, some gypsy women opened my bag. You have to be cautious at every step.
		The confidence I had gained abroad is now shrinking while living in Latvia again, and it makes me feel like I must start over. It feels like my experience abroad has no value here, that I’m not needed here, and that no one appreciates me.

This table presents selected quotes from return migrants, reflecting the factors that either helped or hindered their re-adjustment in Latvia.

#### 3.5.1 Contributing factors

*Freedom and spontaneity*: Many participants highlighted the increased freedom and spontaneity in their daily lives compared to the structured environments of host countries.

*Closeness to nature:* Access to nature and outdoor activities provided a sense of peace and improved their quality of life.

*Economic benefits:* Lower living costs eased the financial burden and enhanced overall satisfaction.

*Cultural and family connections:* Shared traditions, cultural events, and family support strengthened participants’ sense of belonging and provided emotional stability.

*Opportunities for growth:* Some participants discovered personal and professional opportunities that aligned with their ambitions, making the transition smoother.

*Joy in daily life:* Rediscovering joy in everyday activities and the slower pace of life in Latvia contributed to a positive re-adjustment experience.

#### 3.5.2 Disruptive factors

*Bureaucracy:* Rigid and unhelpful administrative systems in Latvia were a source of frustration, with time-consuming processes adding stress to their reintegration.

*Cultural adaptation:* Struggles with adapting to local customs, social norms, and cultural differences often led to feelings of exclusion and disconnection from the community.

*Professional barriers:* Participants experienced challenges in securing meaningful employment, with their international qualifications often undervalued, leading to underemployment or dissatisfaction with available opportunities.

*Societal attitudes:* Narrow-mindedness and perceived lack of openness in Latvian society created barriers to acceptance and reintegration, leaving participants feeling judged or unwelcome.

*Economic challenges:* Low wages in Latvia posed financial difficulties, impacting participants’ ability to maintain the standard of living they had hoped for.

*Environmental factors:* Adjusting to the physical environment, including unfavorable weather and urban aesthetics, contributed to feelings of alienation and disappointment.

## 4 Discussion

The process of return migration presents significant psychological challenges arising from various stress factors that returnees encounter. Although existing literature addresses the psychological aspects of return migration, it often does so in a fragmented manner. This research consolidates these findings by providing a comprehensive overview of the psychological factors affecting return migrants, offering a more cohesive understanding of the critical role psychology plays in the return migration process.

### 4.1 Pre-return context

The pre-return context emerged as an important yet relatively underexplored theme in the study of return migration. Traditionally, research has focused on post-return experiences. According to Pitts (2016) and the Integrative Communication Theory of Cross-Cultural Adaptation (ICCTA) model, the pre-return phase is just as central to return migrants’ narratives as post-return adjustments. This study supports and extends Pitts’s findings by demonstrating that participants’ reflections on their motivations, concerns, and emotional states before returning to Latvia were integral to their overall migration experience.

The literature consistently highlights prior preparation as one of the most critical factors for successful post-return adjustment. Correlational studies have linked the lack of prior preparation to greater difficulties in readjustment (Chamove and Soeterik, 2006). Our findings align with this, showing that participants who took steps to prepare before returning to Latvia generally experienced fewer challenges. Previous migration studies in Latvia have shown that people often emigrate for economic reasons but return for various emotional reasons (Zača et al., 2018). In our study, emotional factors such as proximity to family emerged as significant motivations for return. The COVID-19 pandemic intensified these motivations; emigrants faced travel restrictions that prevented them from visiting family and were concerned about the health and wellbeing of their loved ones. For several respondents, the pandemic was the primary reason for their return, as they perceived Latvia to be a safer place to endure the crisis.

### 4.2 Identity shifts

The exploration of identity in this study closely aligns with Sussman’s (2000) model of cultural identity shifts for return migrants, particularly regarding affirmative, subtractive, and additive identities. In Sussman’s model:

*Affirmative Identity:* Returnees reinforce their home culture connections, feeling a deep sense of belonging upon returning.

*Subtractive Identity:* Returnees experience alienation and disconnection from their home culture.

*Additive Identity:* Returnees integrate elements of both host and home cultures, forming a hybrid sense of self.

Participants who felt a deep sense of belonging upon returning to Latvia reflected the affirmative identity. Those who experienced alienation and disconnection from Latvian society demonstrated the subtractive identity. Participants navigating between host and home cultures mirrored the additive identity, forming a hybrid sense of self. Interestingly, some with an additive identity also exhibited traits of a global identity, suggesting a more fluid and expansive worldview.

Furthermore, this study revealed a distinct category: *identity crisis*. Participants experiencing this crisis struggled with a sense of non-belonging in both their host and home countries, signaling a more intense internal conflict than Sussman’s model accounts for. The concept of identity crisis is closely linked to feelings of detachment and lack of belonging upon returning to one’s home country. Recent literature aligns this identity crisis with newer frameworks, such as the embedding problem in return migration, where individuals struggle to reconcile their prior sense of belonging with the cultural changes experienced abroad (Grabowska and Ryan, 2024). Return migrants often face a disconnect between the new values they adopted while living abroad and the home culture to which they are trying to readjust, resulting in a sense of displacement and internal conflict.

### 4.3 Perceived social support

Perceived social support is essential for mental health, as it helps to mitigate stress and enhance overall wellbeing. Research shows that individuals with higher levels of perceived social support experience less stress, leading to greater positive emotions and reduced symptoms of anxiety and depression (Acoba, 2024). This study’s findings align with existing literature on return migration, which highlights the critical role of family and friends in providing both emotional and practical support to returnees (Van Gorp et al., 2017). Similarly, return migrants in Latvia rely heavily on these networks, emphasizing their importance in the readjustment process. Strong social connections significantly facilitate successful reintegration, while their absence can result in feelings of isolation and heightened stress, complicating the return experience.

In the specific context of Latvia, this study highlights the importance return migrants place on government policies, particularly the role of return migration coordinators. These coordinators often serve as the primary institutional point of contact during the return process, and returnees frequently associate their need for support with the services provided by them. However, a notable observation in this research is the perceived mismatch between the support offered by coordinators and the actual needs of returnees. While coordinators are tasked with offering practical guidance—such as help with documentation, schooling, and housing—many returnees expressed dissatisfaction with the lack of emotional or personalized support. This suggests that return migrants may expect more comprehensive assistance that also addresses their psychological and emotional challenges. This gap indicates a need for Latvian return migration policies to evolve beyond logistical support and consider the holistic wellbeing of returnees. By doing so, institutional support could be better aligned with the comprehensive needs of migrants, contributing more effectively to their reintegration process.

### 4.4 Psychological wellbeing

Psychological wellbeing is defined as a balance of positive emotions, resilience, and meaningful engagement with life, all of which contribute to overall mental health and adaptive functioning (Park et al., 2022). This wellbeing is crucial for return migrants during the reintegration process, as it is often influenced by the gap between their expectations of reentry and the realities they encounter. The Expectations Model (Szkudlarek, 2010) explains the challenges that arise when pre-return expectations clash with post-return realities. While much focus has been placed on outbound expatriation experiences, this model highlights that the complexities of reentry are often overlooked. Many return migrants assume that reintegration into their home country will be seamless, only to face unexpected challenges.

This aligns with the experiences reported in our study, where participants frequently expressed a collision between their pre-return expectations and the realities they faced upon returning to Latvia. In addition to unmet expectations, feelings of alienation and a grieving stage emerged as significant emotional experiences. Many returnees described a sense of not fully belonging either in Latvia or in their previous host country, reflecting a deep sense of disconnection. This form of alienation was often accompanied by the realization that the “home” they returned to had changed, or perhaps they themselves had changed during their time abroad. The sense of alienation can manifest as feelings of isolation, powerlessness, or detachment from the cultural and social environment they once belonged to. Such experiences are often due to the changes that occurred during their absence, leading to challenges in reintegration and adaptation. For instance, a study by Fanari et al. (2021) highlights that return migrants frequently encounter difficulties in re-establishing social connections and aligning with societal norms, contributing to a profound sense of alienation. Alongside alienation, many returnees also experienced a sense of loss, grieving the social networks, professional status, or lifestyle they had built abroad. This emotional burden further complicated their reintegration, as they struggled to reconcile their past experiences with their new realities (Butcher, 2002).

These findings highlight the need for a more comprehensive approach to addressing the psychological wellbeing of return migrants. Distress and anxiety were common themes, with participants frequently expressing stress related to the uncertainty of readjusting to life in Latvia. Much of this anxiety stemmed from concerns about employment prospects and the ability to re-establish social networks. The challenge of balancing personal emotions with the practicalities of reintegration further compounded these feelings. In addition to anxiety, emotions such as guilt and hope played significant roles in shaping participants’ experiences. Guilt often emerged from the pressure to ensure a successful return, particularly regarding family wellbeing, while feelings of hope and excitement surfaced as participants rediscovered familiar cultural aspects of life in Latvia. This complex emotional landscape—ranging from distress and anxiety to guilt, hope, and relief—reveals the profound psychological toll that the return migration process can have (Černigoj et al., 2024; Szkudlarek, 2010).

### 4.5 Factors that help or hinder the readjustment

The factors that help or hinder the readjustment of return migrants can be effectively compared to the push and pull factors often used in migration studies. In return migration, these push and pull dynamics shift: push factors relate to negative experiences abroad, while pull factors focus on what the home country offers that motivates migrants to return (Kļave and Šūpule, 2019). This study demonstrates that family connections, cultural familiarity, and a perceived opportunity for a better quality of life in Latvia serve as strong pull factors.

The push factors identified include feelings of alienation and cultural disconnection abroad, unmet expectations regarding career or lifestyle, and burnout from the fast-paced, competitive environments of host countries. Additionally, a desire for a simpler, more fulfilling life motivated many participants to return to Latvia. Understanding these factors is essential for developing targeted support mechanisms that address both the motivations for return and the challenges faced during reintegration.

In conclusion, this study highlights how crucial psychological factors are for return migrants. It is not just about the practical side of coming back home; successful reintegration deeply depends on how well people prepare beforehand, the shifts in their identity, the support they get from others, and their emotional wellbeing. By focusing on these psychological aspects—especially in countries like Latvia—we can improve policies and support services to better assist returnees. This attention can help make for return migrants’ transition back home smoother and more rewarding.

## 5 Conclusion and implications

This study provides a comprehensive understanding of the psychological dynamics that return migrants face before, during, and after returning to their home country. The findings reveal that alongside practical reintegration challenges such as securing housing and employment, psychological aspects—including identity conflict, social disconnection, unmet expectations, and emotional struggles—are equally significant. These emotional challenges are exacerbated by the interplay of push and pull factors, illustrating how personal, cultural, and institutional dimensions influence the re-adjustment process.

Moreover, the study highlights that current institutional support in Latvia often falls short in addressing the full range of return migrants’ needs, particularly in providing emotional and psychological assistance. Recognizing and leveraging resources and protective factors—such as strong family ties, supportive social networks, cultural familiarity, and personal resilience—can significantly aid the reintegration process. Incorporating these elements into support frameworks can create a more comprehensive and effective approach to assisting return migrants.

The research has several key implications:

- *Holistic support programs:* Government initiatives should integrate psychological support with practical assistance, offering services such as counseling, mental health resources, and support groups alongside help with housing and employment.
- *Training for coordinators:* Return migration coordinators and institutional actors should receive training to identify and address psychological challenges, ensuring they can provide empathetic and comprehensive support.
- *Utilizing protective factors:* Policies should actively incorporate resources and protective factors that facilitate reintegration, such as fostering strong family connections and community networks, which can enhance emotional wellbeing and resilience among return migrants.

Future research should continue to explore the psychological stressors in return migration while also focusing on protective factors that mitigate these challenges. Developing a comprehensive framework that includes both stressors and coping resources will provide a fuller understanding of return migrant experiences. Additionally, comparative studies across different cultural contexts can identify universal and context-specific factors affecting reintegration.

## 6 Strengths and limitations

Corporate repatriates and students are the most researched groups in the field of return migration (Szkudlarek, 2010). Although these populations are easily accessible, they do not represent the most frequent group—self-initiated work-related migrants (Černigoj et al., 2024). For instance, students are generally younger, tend to spend shorter periods abroad, and often return with different expectations compared to self-initiated migrants. Conversely, employees sent abroad on corporate assignments typically know they will return after a fixed period, which can influence their outlook and preparation for reintegration.

In the context of Latvia, understanding the stress factors associated with return migration is crucial for shaping national return migration policies. This research informs outreach efforts aimed at encouraging members of the diaspora to return and supports initiatives to alleviate the stress factors faced by returnees. Additionally, collecting feedback on existing services is vital, such as reevaluating formal barriers to financial aid, as participants indicated that accessing these benefits can be challenging.

One limitation of this study is its focus on a single nation, which may mean that some findings are unique to Latvia and not fully generalizable to other contexts. However, Latvia is a typical country from which many economic migrants have departed, and the emerging themes in this study align with trends observed in similar countries. This suggests that the findings may have broader relevance to other nations with comparable migration histories.

Another limitation is the retrospective nature of this study. Although most participants had returned within the past year at the time of the interviews, the time lag may have influenced how they recall and narrate their experiences. This could affect the accuracy of their recollections. Nonetheless, the proximity to their return enhances the relevance of their insights for understanding the immediate challenges of reintegration.

## Data Availability

The original contributions presented in the study are included in the article/supplementary material, further inquiries can be directed to the corresponding author/s.
